# Molecular Structure, Spectroscopic, Frontier Molecular Orbital Analysis, Molecular Docking Studies, and *In Vitro* DNA-Binding Studies of Osmium(II)-Cymene Complexes with Aryl Phosphine and Aryl Phosphonium Assemblies

**DOI:** 10.1155/2024/6697523

**Published:** 2024-05-29

**Authors:** Kgaugelo C. Tapala, Nqobile G. Ndlangamandla, Mpho P. Ngoepe, Hadley S. Clayton

**Affiliations:** Chemistry Department, University of South Africa, Unisa Science Campus, Johannesburg 1709, South Africa

## Abstract

X-ray crystallography, spectroscopy, computational methods, molecular docking studies, and *in vitro* DNA-binding studies have been useful in the investigations of intermolecular and intramolecular interactions of osmium-cymene oxalato complexes with aryl phosphine and aryl phosphonium groups in both primary and secondary coordination spheres, respectively. Molecular structures of the novel complexes PPh_4_[Os(*η*^6^-*p*-cymene)Br(*κ*^2^-*O,O′*-C_2_O_4_)] (**1**) and [Os(*η*^6^-*p*-cymene) (*κ*^2^-*O,O′*-C_2_O_4_)PPh_3_] (**2**) were resolved by single-crystal X-ray diffraction (XRD). Primary and secondary coordination sphere contacts were investigated using Hirshfeld surface analysis which was supported by molecular docking (MD) studies. The MD data obtained predicted significant differences in binding energy across three receptors for the two osmium complexes. An *in vitro* DNA-binding study was accomplished using UV-Vis spectroscopy which showed that both **1** and **2** bond with DNA through an intercalation approach. The optimized molecular geometry, frontier molecular orbital (E_HOMO_ and E_LUMO_) energies, global electrophilicity index (*ω*), chemical hardness (*η*), chemical potential (*µ*), and the energy band gap (E_HOMO_–E_LUMO_) were calculated utilizing density functional theory (DFT) methods. Computed structural parameters (bond lengths and angles) support the experimental single-crystal XRD data.

## 1. Introduction

**Figure 1:**
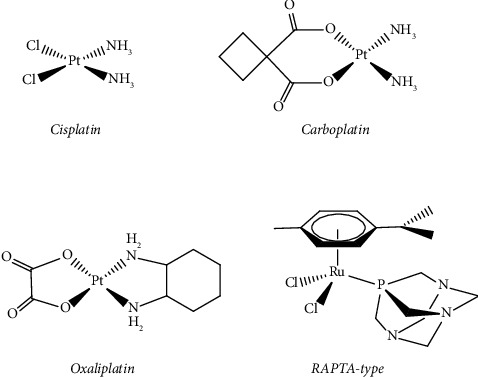
Platinum- and ruthenium-based anticancer metallodrugs.

Within cancer chemotherapy treatment, platinum-based complexes, such as *Cisplatin*, *Carboplatin*, and *Oxaliplatin*, have achieved remarkable success and constitute one of the best extensively utilized classes of metallodrugs [1–5]. These complexes have a similar chemical structure, effecting a square planar geometry with the central Pt(II) ion bearing a pair of N-donor moieties as the stable or nonleaving group ligands and two labile ligands such as halides or an *O,O′*-chelator (Figure 1). However, despite the success of these metallodrugs in treating cancer, their clinical application is constrained by undesirable effects, specifically neuro-, hepatic-, and nephrotoxicity as well as inherent or acquired resistance. To overcome the limitations of platinum-based metallopharmaceuticals, numerous research initiatives to find more metal complexes, which have potential applications in cancer treatment, have improved significantly in the area of medicinal inorganic chemistry [6–8]. Most notable is the development of ruthenium-based RAPTA-type complexes that have 1,3,5-triaza-7-phosphaadamantane (PTA) group, including the corresponding osmium analogues, which both exhibit promising anticancer properties [9–12].

The oxalato ligand performs a critical function in the anticancer activity of the platinum-based drug, *Oxaliplatin*. The *O,O′*-chelator remains bound to platinum *in vivo* until the complex enters the cell cytoplasm, where the oxalato ligand is then replaced by chloride ligands to activate the complex [1, 13]. The activated complex can then covalently bind to the imidazole N7 of guanine. Hydrogen bonding interactions have been found to perform a critical task in the stabilization of the metal-DNA adducts formed by the activated complex [14]. However, when bound to a different metal, such as osmium, the oxalato moiety could function as an intercalating agent, inducing conformation changes to the DNA, and consequently disrupting replication and transcription.

In coordination complexes, ligands connected directly to the metal centre comprise the primary coordination while ligands which are not directly linked but are bound to the metal through noncovalent interactions comprise the secondary coordination sphere. The outer coordination sphere can be manipulated through ligand modification to direct the reactivity of the metal complex and has also been shown to be key in the functioning of metalloproteins [15]. The secondary coordination sphere in metalloproteins is controlled by weak electrostatic forces and plays a key role in molecular recognition as well as in influencing the reactivity and stability of the molecule [16, 17].

Hirshfeld surface analysis is rapidly gaining traction in molecular structure research. This tool offers novel and key understanding of the intermolecular interactions in molecular crystals. Hirshfeld surfaces are particularly valuable for complexes where the surface morphology is not just a consequence of intermolecular packing but also reflects the delicate balance of forces between individual atoms within the molecules, making them powerful tools for deciphering the intricate interplay of these interactions within the crystal lattice [18]. Besides the visual map, fingerprint plots offer a quantifiable breakdown of the different intermolecular relations, revealing their relative influences concerning the molecule's stability [19].

Molecular docking analysis is a key tool for simulating the interactions between proteins and transition metal complexes [20]. This computational tool gives information about the potential for binding between proteins and complexes, the binding energies, the binding positions on the protein, and the nature of interactions [21].

DNA is the key carrier of genetic material and has been broadly investigated as a primary target for numerous metallodrugs. Generally, the binding of metal complexes to DNA arises *via* three approaches of connecting that is: intercalation, electrostatic attraction, and groove binding [22–26]. Therefore, an *in vitro* DNA binding study is key to understanding interactions with metal compounds [27]. The most widely used method for probing *in vitro* DNA binding with metal complexes is with electronic absorption spectroscopy [28, 29].

**Scheme 1:**
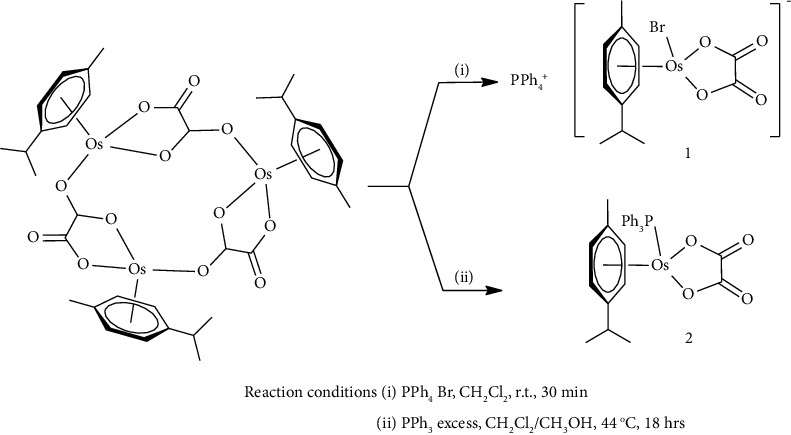
Synthetic pathways of complexes **1** and **2**.

This study investigates the influence of primary and secondary coordination sphere phosphine and phosphonium groups on the vibrational spectroscopy, molecular docking, and hydrogen bonding contacts of osmium complexes PPh_4_[Os(*η*^6^-*p*-cymene)Br(*κ*^2^-*O,O′*-C_2_O_4_)] **(1)** and [Os(*η*^6^-*p*-cymene) (*κ*^2^-*O,O′*-C_2_O_4_)PPh_3_] **(2)** (Scheme 1). For the complexes reported in this project, the designed and prepared Os(II) complexes contain structural features found in the platinum and ruthenium-based metallodrugs cited above. In this study, the structural motif of the PTA ligand has been emulated by the triphenylphosphine ligand which has been shown to enhance the hydrophobicity of the metal-arene complex, effecting increased levels of cytotoxicity *in vitro* [30].

In our study, the osmium complexes were docked against the human serum transferrin, human serum albumin, and DNA duplex. The proteins were selected based on their involvement in cancer tumour growth or their role as transporting agents that influence drug movement within the body. In addition, the molecular structures have been resolved utilizing single-crystal X-ray diffraction while the molecular docking findings were confirmed through conducting *in vitro* DNA-binding studies for complexes in this study.

## 2. Materials and Methods

### 2.1. General Comments

The synthesis methods in this study were all prepared in the presence of an argon atmosphere employing standard Schlenk techniques. Triphenylphosphine (PPh_3_, CAS number 603-35-0, purity ≥99%) and tetraphenylphosphonium bromide (PPh_4_Br, CAS number 2751-90-8, purity ≥97%) were secured from Sigma-Aldrich and were utilized as received. Dichloromethane (CH_2_Cl_2_, CAS number 75-09-2, purity ≥99.8%) from Sigma-Aldrich was dried over calcium hydride (CaH_2_, CAS number 7789-78-8, purity ≥95%) and distilled under argon. Methanol (CH_3_OH, CAS number 67-56-1, purity ≥99.8%) from Sigma-Aldrich was dried over magnesium (Mg, CAS number 7439-95-4, purity ≥98%) and iodine (*I*_2_, CAS number 7553-56-2, purity ≥99.8%) and distilled under argon. Hexane (CH_3_CH_2_CH_2_CH_2_CH_2_CH_3_, CAS number 110-54-3, purity ≥95%), diethyl ether (CH_3_CH_2_OCH_2_CH_3_, CAS number 60-29-7, purity ≥99.0%), and dimethyl sulfoxide (DMSO, CAS number 67-68-5, purity ≥99.9%) were procured through Sigma-Aldrich and were utilized as received. Activated Calf Thymus DNA (CAS number 27-4575-01) was purchased from Sigma-Aldrich with initial concentration of 25 mg/mL. Tris(hydroxymethyl)aminomethane (CAS number 77-86-1, purity ≥99.8%) was procured from Sigma-Aldrich.

### 2.2. Instrumentations

The solid-state IR spectroscopy data were collected from a Bruker Vertex 70 Fourier Transform-Infrared instrument employing an attenuated total reflectance (ATR) element, with a resolution of 2 and 32 number of scans with a range of 4,000–400 cm^−1^. Raman spectral data were collected on a Bruker Raman II instrument with a resolution of 4 and 128 number of scans in the range of 5000–0 cm^−1^. UV-Visible spectroscopy information was collected using a Shimadzu UV-Vis 1800 instrument between wavelength range 250 and 800 nm. Melting point data were collected from Mettler Toledo MP50 Melting Point System.

Nuclear Magnetic Resonance (NMR) information was obtained from 500 MHz Agilent Technologies instrument. Tetramethyl silane (SiMe_4_) was used as an external reference standard for both ^1^H and ^13^C NMR studies, whereas for ^31^P studies, phosphoric acid (H_3_PO_4_) was employed for the external reference standard. The Agricultural Research Council-Institute for Soil, Climate and Water (ARC) used the Carlo Erba NA 1500 (Nitrogen, Carbon and Sulfur) to collect microanalysis data.

Diffraction data for the molecular structures of **1** and **2** were obtained from a Bruker D8 Venture Photon CCD area detector diffractometer at 173(2) K. Data reduction was executed by *SAINT-Plus* and XPREP and structure solutions were solved by SHELXS97 [31]. Structure refinements were performed using SHELXL2014/7 [32] and molecular graphics were performed by *ORTEP* for Windows [33] while *WinGX* publication routine software [33] was used to prepare material for publication.

### 2.3. Synthesis Methods

#### 2.3.1. Synthesis of PPh_4_[Os(*η*^6^-P-cymene)Br(*κ*^2^-O,O′-C_2_O_4_)] **(1)**

The precursor [Os(*η*^6^-*p*-cymene)(C_2_O_4_)]_3_ (0.507 g, 0.84 mmol) was suspended in dichloromethane (50 mL). To the above solution, PPh_4_Br (0.426 g, 1.02 mmol) in dichloromethane (20 mL) was added. The combination was blended for 30 minutes at ambient temperature. A solution (yellow) obtained was concentrated to approximately 20 mL *in vacuo*. Hexane (40 mL) was charged to the solution above, and the solution was agitated to afford a suspension, which was purified with hexane (20 mL). The precipitate was separated and vacuum dried. Yield 74% (0.522 g, 0.63 mmol), mp decompose >198.7°C. ^1^H NMR (500 MHz, CDCl_3_): 1.27 (d, 6H, CH(CH_3_)_2_, *J*_(*HH*)_ = 6.8 Hz); 2.23 (s, 3H, CH_3_C_6_H_4_); 2.80 (sept, 1H, CH(CH_3_)_2_, *J*_(*HH*)_ = 6.9 Hz); 5.64 (d, 2H, *η*^6^-C_6_H_4_, *J*_(*HH*)_ = 5.9); 5.91 (d, 2H, *η*^6^-C_6_H_4_, *J*_(*HH*)_ = 5.4 Hz); 7.57–7.91 (m, 20H, PPh_4_). ^13^C{^1^H} NMR *δ*: 18.91 CH_3_C_6_H_4_; 23.01 CH(CH_3_)_2_; 31.66 CH(CH_3_)_2_; {117.82, 117.15, 71.76, 68.86} *η*^6^-C_6_H_4_; 130.79–135.75 C_Aromatic_; 166.90 (CO). ^31^P{^1^H} NMR *δ*: 24.06 (s, PPh_4_). IR (ATR, Diamond; cm^−1^): *ν*_asym(OCO)_ 1695 s/1674 s/1653s; *ν*_sym(OCO)_ + *ν*_(CC)_ 1483w/1436m; *ν*_sym(OCO)_ 1378s/1317w; *ν*_(CC)_ 910vw/886vw; *ν*_(CC)_ + *δ*_(OCO)_ 787s; *ν*_(OsO)_ + *ν*_(CC)_ 530 s/521s. Microanalysis (%) for C_36_H_34_BrO_4_OsP: Theoretical—C 52.23, H 4.26; Obtained—C 52.26, H 4.46. Diffusion of diethyl ether into the complex's solution of dichloromethane produced crystals appropriate for single-crystal X-ray diffraction.

#### 2.3.2. Synthesis of [Os(*η*^6^-p-cymene) (*κ*^2^-O,O′-C_2_O_4_)PPh_3_] **(2)**

The precursor [Os(*η*^6^-*p*-cymene) (C_2_O_4_)]_3_ (0.354 g, 0.59 mmol) with surplus triphenylphosphine (PPh_3_) (0.570 g, 2.17 mmol) were positioned in a Schlenk tube. The solids were suspended in dichloromethane/methanol (1 : 1 20 mL) mixture. The resultant solution (orange-yellow) was refluxed at 44°C with stirring overnight. A solution (yellow) was obtained and cooled to ambient temperature and then filtered. An oil (yellow) was obtained subsequently stripping off the solvent *in vacuo*. The oil (yellow) was cleansed with hexane over 24 hours. A precipitate (yellow) was separated by percolation and vacuum dried. Yield 79% (0.316 g, 0.47 mmol), mp 217.5–219.9°C. ^1^H NMR (500 MHz, CDCl_3_) *δ*: 1.16 (d, 6H, CH(CH_3_)_2_, *J*_(*HH*)_ = 6.9 Hz); 2.01 (s, 3H, CH_3_C_6_H_4_); 2.47 (sept, 1H, CH(CH_3_)_2_, *J*_(*HH*)_ = 6.8 Hz); 5.29 (d, 2H, *η*^6^-C_6_H_4_, *J*_(*HH*)_ = 5.4 Hz); 5.51 (d, 2H, *η*^6^-C_6_H_4_, *J*_(*HH*)_ = 5.9 Hz); 7.41–7.50 (m, 15H, Ph). ^13^C{^1^H} NMR *δ*: 18.04 CH_3_C_6_H_4_; 22.70 CH(CH_3_)_2_; 31.00 CH(CH_3_)_2_; {98.82 (*J* = 3 Hz), 88.39, 79.35 (*J* = 5 Hz), 79.10 (*J* = 4 Hz)} *η*^6^-C_6_H_4_; 128.91–134.40 C_Aromatic_; 164.22 CO. ^31^P{^1^H} NMR *δ*: 1.78 (s, PPh_3_). IR (ATR, Diamond; cm^−1^): *ν*_asym(OCO)_ 1706sh/1693 s/1669s; *ν*_sym(OCO)_ + *ν*_(CC)_ 1482vw/1433w; *ν*_sym(OCO)_ 1371sh/1363sh/1356s; *ν*_(CC)_ 904vw/875w; *ν*_(CC)_ + *δ*_(OCO)_ 786s; *ν*_(OsO)_ + *ν*_(CC)_ 530vs; *ν*_asym(Os-P)_ 510s, 495m; *ν*_sym(Os-P)_ 437m. Microanalysis (%) for C_30_H_29_O_4_OsP: Theoretical—C 53.40, H 4.33; Obtained—C 53.40, H 4.24. Diffusion of diethyl ether into complex's dichloromethane solution produced crystals appropriate for single-crystal XRD.

### 2.4. Molecular Hirshfeld Surfaces Calculations

Hirshfeld surface plots of complexes **1** and **2** were created using Crystal Explorer 17 [34–36]. Hirshfeld surfaces were utilized to establish intermolecular contacts involving H⋯H, H⋯Br and H⋯O contacts. Three-dimensional (3D) Hirshfeld surface diagrams were produced with the d_norm_ (normalized for the atom size) surfaces mapped over a static red-, white-blue colour system signifying short interactions, van der Waals interactions, and longer interactions sequentially. The typical 0.6–2.6 Å view was utilized to create the two-dimensional (2D) fingerprint maps, with the plot axes displaying *d*_*e*_ and *d*_*i*_ distance scales. Two-dimensional fingerprint plots for **1** and **2** were defined for several contact types, including the H⋯H, C⋯H, and O⋯H contacts, to evaluate and illustrate the influence of polar and nonpolar contacts towards the crystal packing forces.

### 2.5. Computational Experimental Section

All the computations were computed by DMol^3^ DFT program as employed in the Accelrys Material Studio® version 2018 software package [37, 38]. All geometry optimizations were accomplished using the nonlocal generalized gradient approximation (GGA) utilizing the Perdew–Burke–Ernzerhof (PBE) exchange-correlation functional [39]. In this study, core electrons of the Os were taken into consideration using a DFT semi-core pseudopotential in conjunction with double numeric, polarised split valence (DNP) basis set. While the DNP basis set is equivalent in size to the Gaussian 6–31 G^*∗∗*^ basis set, the DNP is utmost exact [40]. Optimizations of the geometries were done with unrestricted spins and no symmetry constraints. These optimizations' convergence criterion included the following threshold values: a self-consistent field density convergence threshold of 1 × 10^−5^ Ha was provided, whereas the following values were given for energy, gradient, and displacement convergence: 2 × 10^−5^ Ha, 0.004 HaÅ^−1^, and 0.005 Å, respectively. To authenticate the nature of the stationary positions, a comprehensive frequency analysis using the equivalent theoretical level (GGA/PBE/DNP) was performed on all optimized geometries. The absence of imaginary frequencies was a characteristic of the optimized geometries.

### 2.6. Molecular Docking Study

The rigid molecular docking studies on the osmium complexes were conducted following a method described by Atlam and co-workers [41] employing Hex 8.0 software [41]. The structural coordinates of the complexes were obtained from the crystallographic information files (CIF Files) and then geometrically optimized by GGA/PBE/DNP using DMol^3^ density functional theory (DFT) software, followed by converting the file format to PDB. The structure of the receptors, human serum transferrin (PDB ID: 1D3K), DNA duplex (PDB ID: 1XRW), and human serum albumin (PDB ID: 1H9Z) were obtained from Protein Data Bank (https://www.pdb.org/pdb/home/home.do). All co-crystallized water molecules, ligands, and co-factors were eliminated from the protein structure before molecular docking computations were embarked on with the studied complexes. In the molecular docking calculation, molecules were displayed using 3D parametric functions that determine both surface shape and electrostatic charge. The docked poses were visualized using Discovery Studio 2020.

### 2.7. Assessment of DNA-Binding Activity by UV-Visible Spectroscopy

Electronic absorption spectra for **1** and **2** were analysed in DMSO using the range of 250-800 nm. Stability studies for both complexes using UV-Vis spectroscopy were conducted over the period of 3 hours, at 15-minute intervals, to examine the activities and stability of the **1** and **2** in the chosen solvent system (DMSO and Tris buffer) prior to performing DNA titrations. The DNA-binding studies of reported complexes were accomplished using tris (hydroxymethyl) aminomethane buffer (5 mM Trizma base, 50 mmol NaCl, pH 7.2). The DNA stock solution was produced by diluting 200 microlitres of CT-DNA in 10 mL of tris buffer solution. Molarity of CT-DNA was recorded spectrophotometrically at UV_260_, using molar absorptivity (ɛ_260_ = 6600 M^−1^ cm^−1^) [22, 28, 42] and was found to be 6.20 × 10^−5^ M. The DNA stock solution was preserved in a freezer below 15°C and used in less than 96 hours. Stock solutions of reported complexes in dimethyl sulfoxide were made and diluted further using the buffer to the necessary concentration (1 × 10^−4^ M) [42]. To regulate possible interactions of CT-DNA with the complexes, a fixed concentration of the compounds were used, with varying increments of DNA stock solution being augmented to the sample and reference chambers, to eradicate possible absorption of free CT-DNA [28]. The combination was nurtured for 15 minutes ahead of the analysis of absorption spectra at ambient temperature.

## 3. Results

### 3.1. Vibrational Spectroscopy

The uncoordinated oxalato anion adopts a nonplanar conformation with approximate *D*_2*d*_ point group symmetry which has the irreducible representation given by Γ = 3*A*1 + *B*1 + 2*B*1 + 3*E*. However, upon coordination as a bidentate ligand (*κ*^2^-*O,O′*-C_2_O_4_), a planar conformation is adopted and the symmetry of the oxalato ligand is reduced to *C*_2*v*_ where the irreducible representation is given by Γ=6*A*_1_+2*A*_2_+5*B*_1_+2*B*_2_. In this case, the Raman and Infrared modes were all active [43, 44].

The CO symmetric and asymmetric stretching bands of the carboxylate groups were found in the ranges 1 500–1 400 cm^−1^ and 1 700–1 500 cm^−1^, respectively [45]. In this study, complexes **1** and **2** show some differences in the CO stretching bands which may be attributed to the complex charge and the ancillary ligand. However, both Infrared and Raman bands of **1** and **2** exhibit some inclusions with the occurrence of near-coincidence, which is associated with the *C*_2*v*_ point group of the oxalato ligand [46]. Despite the similarities in spectral appearances, we have observed some differences in the peak splitting in the Infrared and Raman data.

#### 3.1.1. Vibrational Spectroscopy of COO Bands

The Infrared and Raman bands of complexes **1** and **2** were assigned with reference to other previously reported transition metal oxalato complexes [47–55]. The Infrared (2000–400 cm^−1^) and Raman data (2000–0 cm^−1^) for **1** and **2** are presented in Supplementary Figures S1-S4 in the supplementary data section and the proposed assignments are reported in Table 1. The IR data of **1** and **2** exhibit strong stretching bands at 1695s, 1674s, 1653 s cm^−1^ and 1706sh, 1693s, 1669s respectively, which have been assigned to the *ν*_asym_(OCO) mode. The Raman spectra exhibit weak bands for this stretching frequency in both **1** and **2** which correlate with the IR data. The stretching bands assigned as *ν*_sym_(OCO) + *ν*(CC) mode in the Infrared spectra appeared at 1483w, 1436m cm^−1^ and 1482w, 1467vw, 1433m cm^−1^ for **1** and **2** successively. Corresponding Raman vibrational modes were observed at 1483vw, 1460vw, 1440vw cm^−1^ for **1** and 1483vw, 1455vw, 1440vw cm^−1^ for **2**. The symmetric *ν*(OCO) bands in the Infrared spectra of **1** were observed at 1378s/1317w cm^−1^ as both strong and weak bands, whereas for **2** these bands are observed as a single strong band with two shoulder bands at 1371sh, 1363sh, and 1356s cm^−1^. Raman spectra show both weak and medium bands for **1** at 1384m, 1317vw cm^−1^; however, a single weak vibration at 1364w cm^−1^ was detected for **2** corresponding to the *ν*_sym_(OCO) mode. The mode of coordination of the *O,O′*-chelating ligand in these complexes is consistent with a ∆ value > 200 cm^−1^ where ∆ = *ν*_asym_(OCO) − *ν*_sym_(OCO) [56]. The coordination mode of the oxalato ligand in **1** and **2** was additionally supported by X-ray diffraction information.

The *ν*(CC) mode for **1** was observed as three bands in the Infrared spectrum at 910vw, 886vw, and 859vw cm^−1^. Two signals at 904vw and 875w cm^−1^ observed for **2** were assigned as the *ν*(CC) mode for this complex. The Raman spectra showed very weak single bands for both **1** and **2** at 907vw and 889vw cm^−1^ for the *ν*(CC) mode. Weak and strong bands at 808w and 787s cm^−1^ in the IR spectra for **1** was recognised as *ν*(CC) + *δ*(OCO), whereas for **2** these peaks coalesce to a single strong band at 786s cm^−1^ attributed to electronic effects of ancillary ligands. Raman spectra exhibit a medium intensity band corresponding to the *ν*(CC) + *δ*(OCO) mode at 807m cm^−1^ for **1** and 797m cm^−1^ for **2**. Stretching bands were designated as *ν*(OsO) + *ν*(CC) for **1** at 530vs, 521vs cm^−1^ which for **2** similarly coalesce to a single band at 530vs cm^−1^. The *ν*(OsO) + *ν*(CC) mode in the Raman data shows a band at 537vw cm^−1^ for **1** whereas at 534w cm^−1^ for **2**.

#### 3.1.2. Vibrational Spectroscopy of M-P, M-O, and M-X Bands

The comparison of **1** and **2** indicates that the Infrared vibrations at 510s and 495m cm^−1^ can be attributed to *ν*_asym_(Os-P) which, in contrast, exhibited as a very weak band at 495vw cm^−1^ in the Raman data. The symmetric bands *ν*_sym_(Os-P) assigned at 437m cm^−1^ in the IR showed a corresponding broad weak band at 437w cm^−1^ in the Raman spectrum. Furthermore, the Raman spectra show the Os–O bands at 415sh, 391m cm^−1^ for **1**, and 405m cm^−1^ for **2** (see Table 1) [57]. The influence of intramolecular interactions in **2** may account for the alteration of the Os–O medium band by *ca.* 15 cm^−1^ towards higher energies than that observed at *ca.* 390 cm^−1^ for **1**. The Os–Br band in **1** is found within the expected range in literature [58].

### 3.2. Multinuclear Magnetic Resonance Spectroscopy

The ^1^H NMR data of **1** and **2** agree with the proposed structures. Two pairs of doublets attributed to the *p*-cymene aromatic protons appear for complex **1** at *δ* 5.94 and 5.67 ppm. These two sets of doublets for **2** appear at *δ* 5.54 and 5.32 ppm. Surprisingly, these ^1^H signals of **2** are shielded relative to **1** despite the negative charge on the latter complex ion. The reduced electron density in the *p*-cymene ring is attributed to the *π*-acceptor properties PPh_3_ group.

The ^13^C resonance peaks on the ring of the *p*-cymene ligand of **1** were observed in the range of *δ* 89.05-68.86 ppm, whereas for **2** these signals were found in the range of 98.56-78.93 ppm. As expected, **1** and **2** exhibit different oxalato CO signals, at *δ* 166.89 and 164.11 ppm, respectively, due to changes in the ligand system of the two complexes.

The ^31^P NMR information of **1** and **2** further confirmed the positions of the PPh_4_^+^ and PPh_3_ groups in the outer sphere and inner sphere, respectively. Complex **1** gave a ^31^P signal at 24.07 ppm slightly shifted from 23.09 ppm of the PPh_4_Br precursor. Coordination of PPh_3_ to the metal ion was observed to have shifted the ^31^P signal of **2** from –5.53 ppm to 1.78 ppm. In addition, the ^187^Os satellite peaks confirming the direct Os–P bond were observed with *J*(^187^Os–^31^P) = 309 Hz which is consistent with previously reported osmium(II) complexes [59].

### 3.3. Crystallography

#### 3.3.1. Molecular Structure

**Figure 2:**
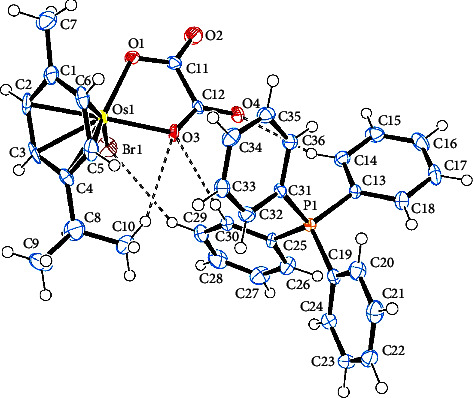
Ortep-3 interpretation of **1** with ellipsoids drawn at the 50% probability level.

**Figure 3:**
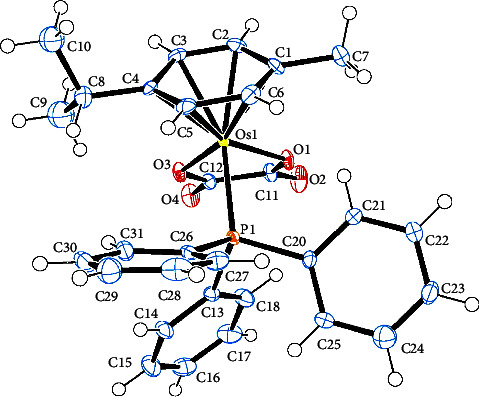
Ortep-3 representation of **2** with ellipsoids drawn at the 50% probability level.

Table 2 contains the crystal data and structural refinement details of complexes **1** and **2**. Molecular structures of **1** and **2** have been elucidated using single-crystal X-ray diffraction (see Figures 2 and 3). Complexes **1** and **2** are both *pseudo*-octahedral, with the hexahaptic *p*-cymene group dominating three coordination positions, and crystalizing in the monoclinic crystal system, with space group P2_1_ (no. 4). The cationic counterion of **1** displays a distorted tetrahedral geometry.

Selected bond lengths, bond, and torsion angles comparing the single-crystal XRD data and DFT-calculated geometrical parameters of complexes **1** and **2** are included in Table S1. The Os–Br bond distance in **1** at *ca.* 2.53 Å is within the range of terminal bromide ligands in previously reported Os(II)-arene complexes [59–61]. The Os–O bond distances in **1** were measured at 2.099(4) Å and 2.100(4) Å, whereas the corresponding Os–O bond distances in **2** were determined to be 2.093(3) Å and 2.078(3) Å. For **2**, the oxalato ligand was found to bind asymmetrically to the osmium centre. This may be due to the larger steric requirement of the PPh_3_ ligand. The Os–P bond distance at *ca.* 2.35 Å observed for **2** is similar to related Os(II) complexes [59–62].

The osmium-cymene centroid distance in **2** was found to be slightly longer than in **1** which was attributed to the inner sphere influence of the osmium bound PPh_3_ ligand. Consequently, the osmium-carbon bond lengths of **1** were found to be marginally shorter relative to **2** due to the sterically demanding coordinated PPh_3_ group. The strong *σ*–donor and *π*–acceptor ability of PPh_3_ effected elongated Os–C bonds *trans* to the P-donor atom. Both **1** and **2** showed loss of aromaticity of the *p*-cymene group evidenced by varying shorter and longer carbon-carbon bond lengths within the ring.

The bite angle O1–Os–O3 of **1** is slightly reduced compared to **2**. This is unexpected as complex **2** would be expected to have a large bite angle due to the steric demand of the large PPh_3_. However, the observed increase in bite angle may be due to electronic factors generated by O-atom lone pair repulsions within the chelate ring of **2**. The combined covalent radii of the Os(II) ion and O donor atom at 2.1 Å are consistent with Os–O single bonds in the metallacycle moiety of both **1** and **2** in this study [63]. Lack of planarity of the metallacycle in both **1** and **2** is indicated by the nonzero torsion angle O1–C11–C12–O3 which is large for **2** because of the steric requirements of the bulky PPh_3_ ligand.

#### 3.3.2. Hirshfeld Surface Calculations

From the XRD data, the structure of **1** shows the PPh_4_^+^ cation and the anionic complex [Os(*η*^6^-*p*-cymene)Br(*κ*^2^-*O,O′*-C_2_O_4_)] that are held jointly *via* two C–H^…^O and one C–H^…^Br interactions. Furthermore, one C–H^…^O intramolecular hydrogen bond exists (see Figure 2). This C–H…O contact defined by the osmium coordinated oxygen atom and the methine hydrogen of the p-cymene isopropyl group was measured with a distance of 2.673 Å and an angle of 149.01°. The C–H^…^O intermolecular hydrogen bond observed links at the same O atom and an H atom of a phenyl group measured 2.479 Å at 149.22°. The second C–H^…^O intermolecular hydrogen bond observed between a carbonyl O atom and a hydrogen of the phenyl group measured 2.582 Å at 145.22°. The third C–H^…^Br intermolecular hydrogen bond detected between the Br atom and a hydrogen of the phenyl substituent measured 2.996 Å at 150.08°. The latter intermolecular hydrogen bonding is classified as a weaker hydrogen bond.

Complex **2** shows a C–H^…^O intramolecular hydrogen bonding owing to one of the O atoms coordinated to the Os metal centre and a C(sp^2^)–H group of one of the phenyl groups measured 2.808 Å at 128.39° (see Figure 3). This interaction is considered a weak contact because of the long range and the angle which deviates significantly from linearity [64]. A second intramolecular hydrogen bond between a metal-coordinated O atom and a C(sp^3^)–H of the propan-2-yl substituent on the *p*-cymene fragment measured 2.545 Å at 133.46°.

**Figure 4:**
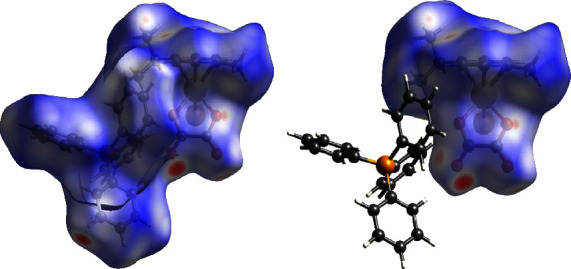
Hirshfeld surfaces for (a) mapping complex **1** and (b) the [Os(*η*^6^ − p − cymene)(*κ*^2^ − O, O′ − C_2_O_4_)] fragment mapped with d_norm_.

**Figure 5:**
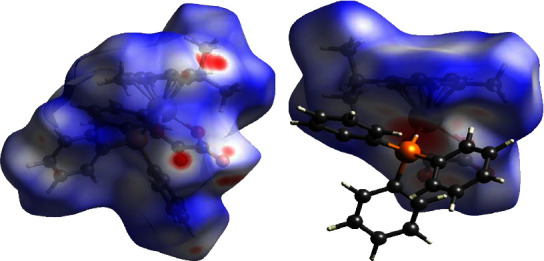
Hirshfeld surfaces for (a) mapping complex **2** and (b) the [Os(*η*^6^−p − cymene)Br(*κ*^2^ − O, O′ − C_2_O_4_)] fragment mapped with d_norm_.

To obtain a greater understanding into the effect of intermolecular forces on the geometry of the osmium complexes, a comparative Hirshfeld surface study of the two complexes was conducted. Hirshfeld surfaces mapped over d_norm_ functions of **1** and **2** are presented in Figures 4 and 5 successively.

In the two-dimensional fingerprint plots, it is understood that the molecule functions as an acceptor if *d*_*i*_ > *d*_*e*_ but the molecule is a donor if *d*_*i*_ < *d*_*e*_. Complex **1** exhibits three short contacts C-H^…^Br, C-H^…^O, and C-H^…^H in XRD. Consistency is observed between the diffraction data and Hirshfeld Surface calculations for **1**. Mapping of **1** as visualized in Figure 4(a) shows several contacts and their contributions (see Figure S5(a) in supplementary data section) were calculated as follows: hydrogen bonds [H⋯H (54.0%), C⋯H (13.8%), O⋯H (7.7%) and Br⋯H (3.7%)], tetrel bonds [C⋯O (0.2%) and C⋯Br (0.2%)] [65]. In addition, the fingerprint plots of osmium-oxalato moiety (see Figure 4(b)) assigned the contributions as follows: hydrogen bonds [H⋯H (45.7%), Br⋯H (9.5%), O⋯H (18.6%), and C⋯H (5.0%)] and tetrel bonds [C⋯C (2.0%), C⋯Br (0.4%), and O⋯C (6.0%)] as seen in Figure S5(b) in the supplementary data section.

Complex 2, as mapped in Figure 5(a), along with the 2-D fingerprint plots given in Fig.  S6 (c), show the computed contacts and the corresponding contributions as follows: hydrogen bonds [H⋯H (58.3%), C⋯H (11.0%), and O⋯H (10.3%)] and a tetrel bond [C⋯C (1.5%)]. The 2-D fingerprint plots of the osmium-cymene oxalato moiety as shown in Figure 5(b) exhibit several contacts (see Figure S6(d) in the supplementary data section) and the fingerprint contributions were determined as follows: hydrogen bonds [H⋯H (51.4%), C⋯H (5.5%), and O⋯H (21.6%)], tetrel bonds [C⋯C (1.8%), C⋯P (0.2%), O⋯C (1.0%)], and a pnictogen bond [O⋯P (0.7%)] [65]. The noncovalent interactions observed in the Hirshfeld surface analysis are consistent with the interactions observed in the packing diagrams of complexes **1** and **2** (see Supplementary Figures S7 and S8 in the supplementary data section).

### 3.4. Theoretical Studies

#### 3.4.1. DMol^3^ Geometry Optimization

The optimized molecular structures, electron density isosurface maps, and the computed energies of the HOMO and LUMO orbitals are presented in Table 3 (optimized geometries and computed FMO). Optimized geometrical parameters accomplished using computation and the experimentally determined data are available in Table 4 (global chemical reactivity indices for 1 and 2). Root mean square (RMS) values of bond lengths, bond angles, and torsion angles are accomplished by employing the following expression:

(1) $$\begin{array}{c} \text{RMS}=\sqrt{\frac{1}{n}\overset{n}{\underset{i}{\sum }}{\left(X_{i}^{\text{cal}.}‐\;X_{i}^{\exp}\right)}^{2}}, \end{array}$$

where *X*^cal.^ and *X*^exp .^ are the computed and the experimental information, in sequence. RMS errors of the bond lengths and internal angles are 0.0918 Å and 0.7412° for complex **1** and 0.0974 Å and 0.6327° for complex **2** successively.

#### 3.4.2. Frontier Molecular Orbital (FMO) Analysis

To gain an understanding of the behaviour of the electronic transitions within the complexes investigated in this study, a quantitative analysis of key quantum chemical parameters of the molecular species was carried out. DFT simulations provided insights into the electronic structures of **1** and **2**, including their energy levels, bonding patterns, and reactivity. Analysing the DFT-derived global reactivity descriptors, particularly the interplay between chemical hardness (*η*) and electrophilicity (*ω*), revealed fascinating insights into the relative stability and reactivity profiles of the complexes. This information provided valuable clues for understanding their potential applications and behaviour.

The frontier molecular orbital (FMO) energies are widespread quantum mechanical descriptors since the orbitals were illustrated to perform the key function towards influencing various chemical reactions. The highest occupied molecular orbital (HOMO) and lowest unoccupied molecular orbital (LUMO) energies are associated to gas phase ionization energies (IP) and electron affinities (EA) according to Koopmans' theorem [66, 67]. The computed reactivity indices are summarized in Table 3.

Chemical hardness (*η*), a concept rooted in frontier molecular orbitals, quantifies a molecule's resistance to changes in its electron distribution or charge transfer. This resistance is directly connected to the energy gap involving the HOMO and the LUMO, as described by equation (2). A wider HOMO-LUMO gap signifies a higher *η*, implying greater stability and reduced reactivity.

(2) $$\begin{array}{c} \eta =\frac{\left(\varepsilon_{\text{LUMO}}\;–\;\varepsilon_{\text{HOMO}}\right)}{2}. \end{array}$$

Designated as the negatively charged electronegativity and computed by equation (3), electronic chemical potential (*μ*) captures the ease with which electrons can escape from a molecule. A higher *μ* indicates a greater tendency for electron loss, making the molecule less stable and more reactive [68].

(3) $$\begin{array}{c} \mu =\frac{\left(\varepsilon_{\text{LUMO}}+\varepsilon_{\text{HOMO}}\right)}{2}. \end{array}$$

Introduced by Parr and calculated using equation (4), the electrophilicity index (*ω*) reflects the molecule's tendency to accept electrons. It combines the stabilizing effect of gaining an electron (*μ*) with the resistance to electron redistribution (*η*), providing a quantitative measure of electron-loving power.

(4) $$\begin{array}{c} \omega =\frac{\mu^{2}}{2\eta }. \end{array}$$

It can be seen from the molecular orbital diagrams in Table 4 that the HOMO and LUMO of **1** are confined to the osmium anionic and phosphonium aryl moieties, respectively, whereas in **2** the HOMO is mainly scattered over the oxalato ligand and the LUMO is extensively dispersed over the osmium-phosphine segment of the complex. The HOMO signifies the dissemination and energy of the least tightly held electrons in the compound while the LUMO identifies the moiety of the compound where the addition of electrons is most probable. Based on the relative HOMO energies of the complexes, it is expected that **1** would more readily donate its electrons and undergo reduction. According to the HOMO-LUMO band gap values, **1** also has the lower excitation energy and would thus exhibit a higher chemical reactivity and lower kinetic stability. A small global hardness (*η*) means that the complex has high polarizability. The smaller *η* value of **1** indicates that the two-component ionic osmium moiety has higher polarizability relative to the neutral complex **2,** which is expected to exhibit greater resistance towards deformation of its electron cloud under small perturbations. The negligible difference in electronegativity indicates that both **1** and **2** have a similar capacity for attracting electrons from the neighbouring molecules. The electronic chemical potential (*µ*) is a property of an equilibrium state which indicates that both complexes have comparable capacities for changes in electron density and are expected to undergo similar electron density flux in an interacting system.

### 3.5. Molecular Docking Studies

Molecular docking results of **1** and **2** are reported in Table 5. The Hex docking results reveal that **1** has an improved binding potential than **2** due to the lower free energy indicating better interaction. Across different receptors, complex **1** showed stable binding energies with an average of −284.1 ± 4.56 kJ/mol while **2** showed variations between different receptors with an average energy of −214.9 ± 57.99 kJ/mol. Thus, **1** was found to have better binding energy than **2** against all the evaluated receptors. Interestingly, no halogen-type interactions were observed with the bromo group in complex **1**. However, the methyl groups and the phosphonium aryl group have contributed mostly to the hydrophobic interaction, while the oxygen atoms play a role in attractive (dipole-dipole) interactions.

**Figure 6:**
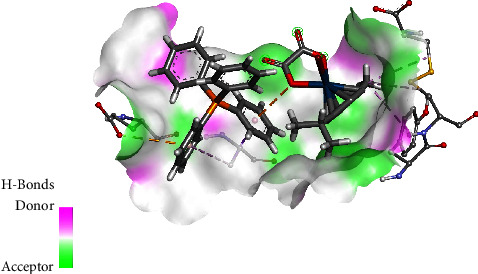
Interaction of **1** with human serum transferrin.

**Figure 7:**
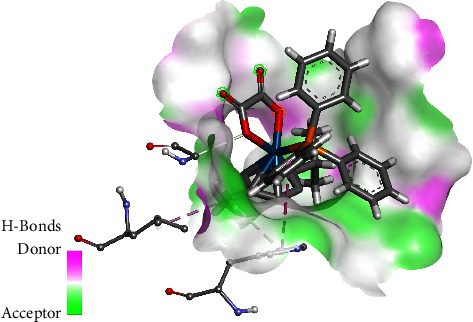
Interaction of **2** with human serum transferrin.

The molecular docking studies of **1** and **2** against human serum transferrin (1D3K) exhibited some occurrences of hydrogen bonds. For complex **1**, the osmium complex formed alkyl-*π* interactions with Cys137(A), Tyr136(A), and Cys331(A) attributed to methyl group of the *p*-cymene, as shown in Figure 6. The oxygen within the chelate ring interacted with one of the phenyl rings on the phosphonium moiety through a *π*-anion interaction. The phosphonium moiety also formed van der Waals interaction with Gly133(A), Asn325(A), Thr321(A), Tyr317(A), Tyr319(A), and Ala244(A). The phenyl ring of PPh_4_^+^ cation also formed *π*-anion interactions with Glu318(A) whilst also playing a role in *π*-*σ* and *π*-alkyl interaction with Ala322(A). For complex **2**, there are strong van der Waals force interactions with various amino acids [Lys291(A), Ser189(A), Gly190(A), Tyr185(A), Phe186(A), Lys193(A), Asn183(A), Gly187(A), Gln184(A), Ser180(A), Leu182(A), and His289(A)]. A carbon-oxygen contact occurred via the oxygen of the coordinated oxalato ligand interacting with Gly290(A), whereas the methyl group of the cymene ligand exhibits a *π*-alkyl contact with Leu293(A) and His14(A) as illustrated in Figure 7. In addition, one of the aromatic rings of the metal-coordinated PPh_3_ exhibited *π*-*π* stacking with His14(A).

**Figure 8:**
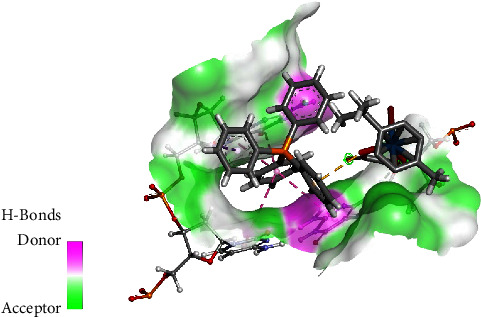
Interaction of **1** with DNA duplex.

**Figure 9:**
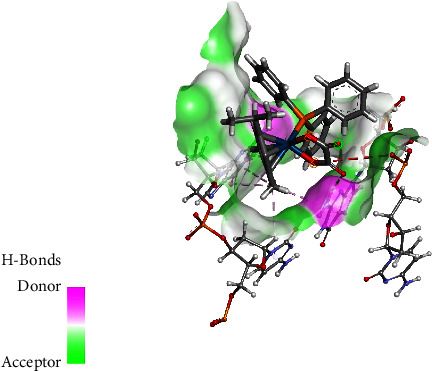
Interaction of **2** with DNA duplex.

The interactions of the complexes were also evaluated against DNA Duplex (1XRW). In complex **1**, the osmium complex moiety formed *π*-anion interaction with the phosphonium moiety through the oxygen, as shown in Figure 8. The oxalato ligand carbonyl group interacted with DG5(A), *via* a carbon-hydrogen bond interaction. The complex also forms van der Waals interactions with DC7(A) and DT6(A). The phosphonium moiety of **1** also participates by having van der Waals interaction with DA6(B), DT6(A), and DC7(A). The phenyl rings of PPh4 play a role in *π*-*π* stacking with DC4(B) and DG5(A) while also interacting via *π*-*σ* interaction with DG5(B). For complex **2**, it was observed that the oxygen of the carbonyl played a role in hydrogen bonding with DG5(A), with a ring oxygen having a negative-negative interaction with DC7(A), as shown in Figure 9. The methyl group formed *π*-alkyl interaction primarily with DC4(B), whereas the PPh_3_ ligand played a role in *π*-*π* stacking with DG5(B).

**Figure 10:**
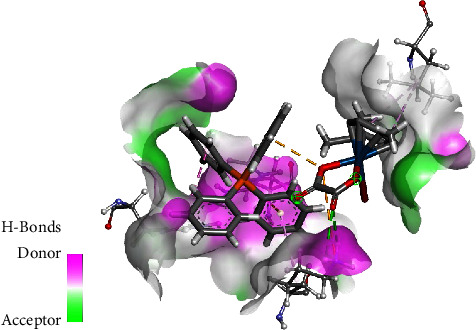
Interaction of **1** with human serum albumin.

**Figure 11:**
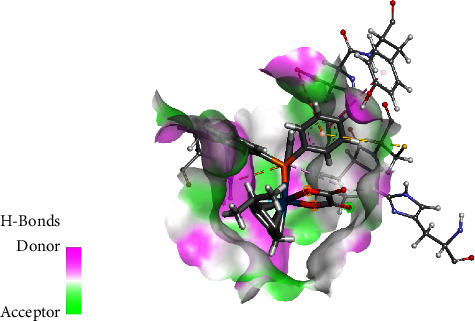
Interaction of complex **2** with human serum albumin.

The interaction of complexes **1** and **2** against human serum albumin (1H9Z) was investigated. In complex **1**, the Os-O moiety was observed to form an attractive charge interaction with Lys190(A) while simultaneously interacting with the phosphonium moiety *via* a *π*-anion interaction, as shown in Figure 10. A hydrogen bond between the oxygen of the carbonyl with Lys190(A) was detected. The methyl substituent on the cymene ring contributed towards *π*-alkyl interaction with Leu463(A). The phosphonium moiety was observed to form a *π*-cation interaction with Arg197(A), while a *π*-*πT*-shaped contact occurred *via* one of the phenyl rings with His146(A) and a second phenyl ring interacted with Lys190(A) via *π*-alkyl interaction. Complex **2** exhibits a carbon-hydrogen interaction with His440(A) via the oxalato carbonyl group. In addition, the PPh_3_ has a *π*-sulfur interaction with Cys448, *π*-*π* T-shaped stack interaction with Tyr452(A), and *π*-anion interaction with Asp451(A), as shown in Figure 11. A second phenyl ring participates in *π*-cation contact involving Arg218(A) and *π*-alkyl interaction involving Pro447(7).

### 3.6. Assessment of DNA-Binding Activity by UV-Visible Measurements

UV-Vis absorption information of **1** and **2** are accessible in the supplementary data section, Supplementary Figures S9 and S10. Complex **1** has four observable absorption peaks, in the range of 250 nm to 350 nm. The signals around 267 nm and 269 nm correspond to n − *π*^*∗*^ electronic transitions; 276 nm corresponds to a *π* − *π*^*∗*^ transition, and 340.25 nm is associated with an MLCT transition of the complex [69]. In comparison, complex **2** has only three peaks appearing at 268 nm, 277 nm, and 340.5 nm consistent to n − *π*^*∗*^, *π* − *π*^*∗*^, and MLCT transitions, respectively [69].

The stability test results for **1** and **2** in the DMSO and Tris buffer binary solvent system are reported in the supplementary data section Supplementary Figures S11 and S12. The data illustrate that both complexes are stable in DMSO and Tris buffer solvent systems, as there were no observable changes over time. There are no ligand exchange reactions or precipitation occurring in the solvent system since a change in the ligation of the metal results in observable changes in the electronic spectrum.

*In vitro* experimentations on the possible interaction of **1** and **2** to DNA were carried out by observing changes in the UV-Vis spectra. When DNA interacts with metal fragments, it could result in hypochromic or hyperchromic changes. Hyperchromic effects are due to an electrostatic binding mode with DNA, whereas hypochromic effects are as a result of an intercalative binding mode [70]. From Supplementary Figures S13 and S14 in the supplementary data section, both complexes show a significant hypochromic effect (decrease in peak intensities). Hypochromic effect is caused by significant damage to the DNA double helical structure, which causes the *π*^*∗*^ orbitals of the ligands in the complex to interact with the *π* orbitals in the DNA base pairs [71]. This results in an enhanced *π* − *π*^*∗*^ stacking assembly involving DNA base pairs with conjugated planar ring systems of the complex [28]. Thus, the resulting coupled *π*^*∗*^ orbitals become partially filled, effecting a decrease in the possible electron transitions [71]. In addition, slight bathochromic changes were detected in the data of both complexes, which has been reported to be due to intercalative bonding of the complex with DNA [28, 42]. It is worth noting that complexes under study, based on the DNA-binding study, show diverse mechanism of action compared to the standard drug, *Oxaliplatin*. Research shows that *Oxaliplatin* bond to DNA covalently by attaching through N7 of the guanine base [13, 72]. Contrarily, the complexes in this study were able to function as an intercalating agent, inducing conformation changes to the DNA, which would disrupt replication and transcription.

## 4. Conclusions

This study provides an insight into the identification of structural parameters that influence intermolecular and intramolecular interactions in complexes of this type, which may assist in the design of new potential metallodrugs. Two novel osmium-cymene complexes containing phosphine as well as phosphonium aryl assemblies in both primary and secondary coordination domains have been prepared and structurally characterized utilizing single-crystal XRD, FT-Raman, FT-IR, UV-Vis, and NMR spectroscopy. From a computational DFT study, HOMO-LUMO orbitals for both complexes have been determined and the relative molecular stability evaluated using FMO analysis. The chemical reactivity descriptors' values highlight that osmium-oxalato complex with the phosphonium aryl moiety in the second coordination sphere exhibits a higher chemical reactivity and lower kinetic stability. The Hirshfeld surface analysis discloses that the transposition of the phosphine aryl group from the inner coordination sphere to a phosphonium aryl group in the outer coordination sphere also has a significant influence on the intra- and intermolecular bonding capabilities of the osmium(II)-oxalato moiety. Hirshfeld surface analysis interactions are supported by molecular docking study results which show several interactions between the complexes and selected receptors. Complex **1** shows lower free energy with stable binding energy across the three receptors compared to complex **2**. Due to the bulkiness and the charged nature of complex **1**, it is more likely to dock in larger pockets and where there are charged species than complex **2**. Complex **2** exhibits variations in binding energies which could be because of its neutral state and hydrophobic nature which leads to interaction with amino acids that favour hydrophobic interactions. The stability of both complexes was evaluated in DMSO and Tris buffer to warrant the use of this solvent system for DNA titrations. From the DNA-binding study, it can be concluded that both **1** and **2** bond to CT-DNA *in vitro*, possibly applying the intercalation approach of binding. The behaviour is attributed to effective *π* − *π*^*∗*^ stacking connections involving the DNA base pairs and conjugated planar ring system chromophores of the complex. These results agreed with *in silico* docking studies that were executed to develop an understanding of the interactions in **1** and **2** against DNA Duplex (1XRW), as both complexes showed possible *π*-*π* interactions with DNA Duplex.

## Figures and Tables

**Table 1:** Assignment of vibrational (Infrared and Raman) data of complexes **1** and **2**.

1		2		Assignments
IR	Raman	IR	Raman	
1695s, 1674s, 1652s	1697w, 1680w, 1654w	1706sh, 1693s, 1669s	1697w, 1669w	*ν*_asym(OCO)_
1483w, 1436m	1483vw, 1460v, 1440vw	1482w, 1467vw, 1433m	1483vw, 1455vw, 1440vw	*ν*_sym(OCO)_+*ν*_(CC)_
1378s, 1317w	1384m, 1317vw	1371sh, 1363sh, 1356s	1364w	*ν*_sym(OCO)_
910vw, 886vw, 859vw	907vw	904vw, 875w	904vw, 889vw	*ν*_(CC)_
808w, 787s	807m	786s	797m	*ν*_(CC)_+*δ*_OCO_
530vs, 521vs	537vw	530vs	534w	*ν*_(OsO)_+*ν*_(CC)_
—	—	510s, 495s	512vw, 495vw	*ν*_asym(Os − P)_
—	—	437m	437w	*ν*_sym(Os − P)_
—	415sh, 391m	—	405m	*ν*_(Os − O)_
—	197w, 176vw	—	—	*ν*_(Os − Br)_

Very strong, vs; strong, s; medium, m; weak, w; very weak, vw; shoulder, sh.

**Table 2:** Crystal data and structural refinement of **1** and **2**.

	1	2
CCDC no.	1909507	1909506
Empirical formula	C_36_H_34_BrO_4_OsP	C_30_H_29_O_4_OsP
Formula weight	831.71	674.70
Crystal system	Monoclinic	Monoclinic
Space group	P2_1_ (no. 4)	P2_1_ (no. 4)
Crystal colour and shape	Yellow sheets	Yellow cubes
Crystal size (mm^3^)	0.528 × 0.368 × 0.083	0.829 × 0.481 × 0.334
*a* (Å)	7.5565 (8)	9.745 (5)
*b* (Å)	10.7431 (12)	11.825 (5)
*c* (Å)	19.144 (2)	11.403 (5)
*α* (°)	90.00	90.000 (5)
*β* (°)	100.154 (4)	99.261 (5)
*γ* (°)	90.00	90.000 (5)
*V* (Å^3^)	1529.8 (3)	1296.9 (10)
*Z*	2	2
*T* (*K*)	173 (2)	173 (2)
*D*_calc_ (mg/m^3^)	1.806	1.728
Absorption coefficient (mm^−1^)	5.566	5.012
Reflections collected	70605	59101
Independent reflections	7361 [*R*_(int)_ = 0.0939]	6222 [*R*_(int)_ = 0.0398]
Data/restraints/parameters	7361/1/391	6222/1/308
*F* (000)	816	664
Final *R* indices [*I* > 2*σ*(*I*)]	*R*1 = 0.0240, w*R*2 = 0.0623	*R*1 = 0.0206, w*R*2 = 0.0529
R indices (all data)	*R*1 = 0.0245, w*R*2 = 0.0636	*R*1 = 0.0210, w*R*2 = 0.0532
Goodness-of-fit on *F*^2^	0.421	0.945
Largest diff. peak and hole (e·Å^−3^)	0.727 and −2.154	0.815 and −2.134

**Table 3:** Optimized geometries and computed FMO.

Optimized structures	LUMO	HOMO
**1**		
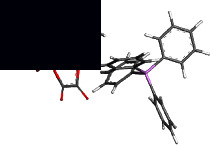	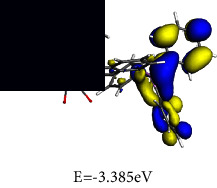	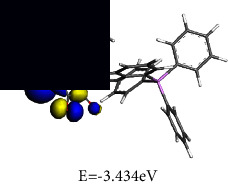

**2**		
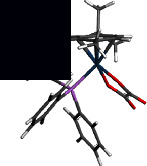	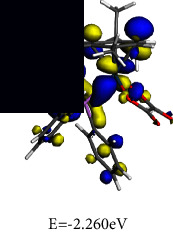	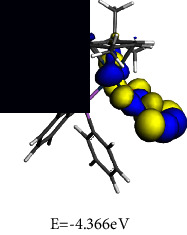

**Table 4:** Global chemical reactivity indices for **1** and **2**.

Molecular properties	DFT calculated values (eV)	
	1	2
IP	3.434	4.366
EA	3.385	2.260
*η*	0.025	1.053
*χ*	3.409	3.313
*μ*	−3.409	−3.313
*ω*	237.239	5.212

**Table 5:** Binding energies of complexes **1** and **2** with receptors showing *E*-value (kJ/mol).

Receptor	1	2
Human serum transferrin (1D3K)	−281.4	−239.3
DNA duplex (1XRW)	−289.4	−256.7
Human serum albumin (1H9Z)	−281.6	−148.7

## Data Availability

Additional crystallography information is accessible without restrictions on the Cambridge Crystallography Data Centre at https://www.ccdc.cam.ac.ac.uk/data and the deposition numbers for the complexes in this study are CCDC-1909507 for **1** and CCDC-1909506 for **2**.

## Supplementary Materials

Supplementary Materials Vibrational spectroscopy data. Figure S1: FT-IR data of **1** from 2000 to 400 cm^−1^. Figure S2: FT-IR data of **2** from 2000 to 400 cm^−1^. Figure S3: FT-Raman spectrum of **1** from 2000 to 0 cm^−1^. Figure S4: Raman spectrum of **2** from 2000 to 0 cm^−1^. Hirshfeld Surface Analysis. Figure S5: Two-dimensional fingerprint plots portrayed into various contact types for complex **1**. Figure S6: Two‐dimensional fingerprint plots portrayed into various contact types for complex **2**. Figure S7: Packing diagram of **1** showing various types of interactions in a unit cell. Figure S8: Packing diagram of **2** showing various types of interactions in a unit cell. Table S1: Single-crystal XRD information and DFT-calculated geometrical parameters of **1** and **2** [Selected bond lengths (Å) and bond and torsion angles (°)]. DNA-Binding Study. Figure S9: UV-Vis absorption data of **1** in DMSO. Figure S10: UV-Vis absorption data of **2** in DMSO. Figure S11: UV-Vis absorbance spectra, illustrating the stability study of **1** in DMSO and Tris buffer over 3 hours. Figure S12: UV-Vis absorbance spectra, illustrating the stability study of **2** in DMSO and Tris buffer over 3 hours. Figure S13: Electronic data of **1** in Tris-HCl buffer following addition of various increments of CT-DNA solution over the period of 3 hours. Figure S14: Electronic data of **2** in Tris-HCl buffer following addition of various increments of CT-DNA solution over the period of 3 hours. X-ray crystallography. CheckCIF/PLATON details for the crystal structure solving of **1** and **2**.
