# Supplementary material for: Molecular Structure, Spectroscopic, Frontier Molecular Orbital Analysis, Molecular Docking Studies, and In Vitro DNA-Binding Studies of Osmium(II)-Cymene Complexes with Aryl Phosphine and Aryl Phosphonium Assemblies
Source: Bioinorg Chem Appl. 2024 May 29;2024:6697523. doi: 10.1155/2024/6697523 (PMC11152764; doi:10.1155/2024/6697523)
Supplement: Supplementary Materials — Vibrational spectroscopy data. Figure S1: FT-IR data of 1 from 2000 to 400 cm−1. Figure S2: FT-IR data of 2 from 2000 to 400 cm−1. Figure S3: FT-Raman spectrum of 1 from 2000 to 0 cm−1. Figure S4: Raman spectrum of 2 from 2000 to 0 cm−1. Hirshfeld Surface Analysis. Figure S5: Two-dimensional fingerprint plots portrayed into various contact types for complex 1. Figure S6: Two‐dimensional fingerprint plots portrayed into various contact types for complex 2. Figure S7: Packing diagram of 1 showing various types of interactions in a unit cell. Figure S8: Packing diagram of 2 showing various types of interactions in a unit cell. Table S1: Single-crystal XRD information and DFT-calculated geometrical parameters of 1 and 2 [Selected bond lengths (Å) and bond and torsion angles (°)]. DNA-Binding Study. Figure S9: UV-Vis absorption data of 1 in DMSO. Figure S10: UV-Vis absorption data of 2 in DMSO. Figure S11: UV-Vis absorbance spectra, illustrating the stability study of 1 in DMSO and Tris buffer over 3 hours. Figure S12: UV-Vis absorbance spectra, illustrating the stability study of 2 in DMSO and Tris buffer over 3 hours. Figure S13: Electronic data of 1 in Tris-HCl buffer following addition of various increments of CT-DNA solution over the period of 3 hours. Figure S14: Electronic data of 2 in Tris-HCl buffer following addition of various increments of CT-DNA solution over the period of 3 hours. X-ray crystallography. CheckCIF/PLATON details for the crystal structure solving of 1 and 2. [file 6697523.f1.zip › Bioinorganic Chemistry and Applications Supplementary Data_April 2024.docx]

**Molecular Structure, Spectroscopic, Frontier Molecular Orbital Analysis, Molecular Docking Studies and *In Vitro* DNA Binding Studies of Osmium(II)-Cymene Complexes with Aryl Phosphine and Aryl Phosphonium Assemblies**

Kgaugelo C. Tapala^1^, Nqobile G. Ndlangamandla^1^, Mpho P. Ngoepe^1^, and Hadley S. Clayton^1^*

^1^Chemistry Department, University of South Africa, Unisa Science Campus, Johannesburg, 1709, South Africa

**Supplementary data**

**Vibrational spectroscopy data**

**Figure S1**: FT-IR spectrum of **1** from 2000 to 400 cm^-1^.

**Figure S2**: FT-IR spectrum of **2** from 2000 to 400 cm^-1^.

**Figure S3**: FT-Raman spectrum of **1** from 2000 to 0 cm^-1^.

**Figure S4**: Raman spectrum of **2** from 2000 to 0 cm^-1^.

**Hirshfeld Surface Analysis**


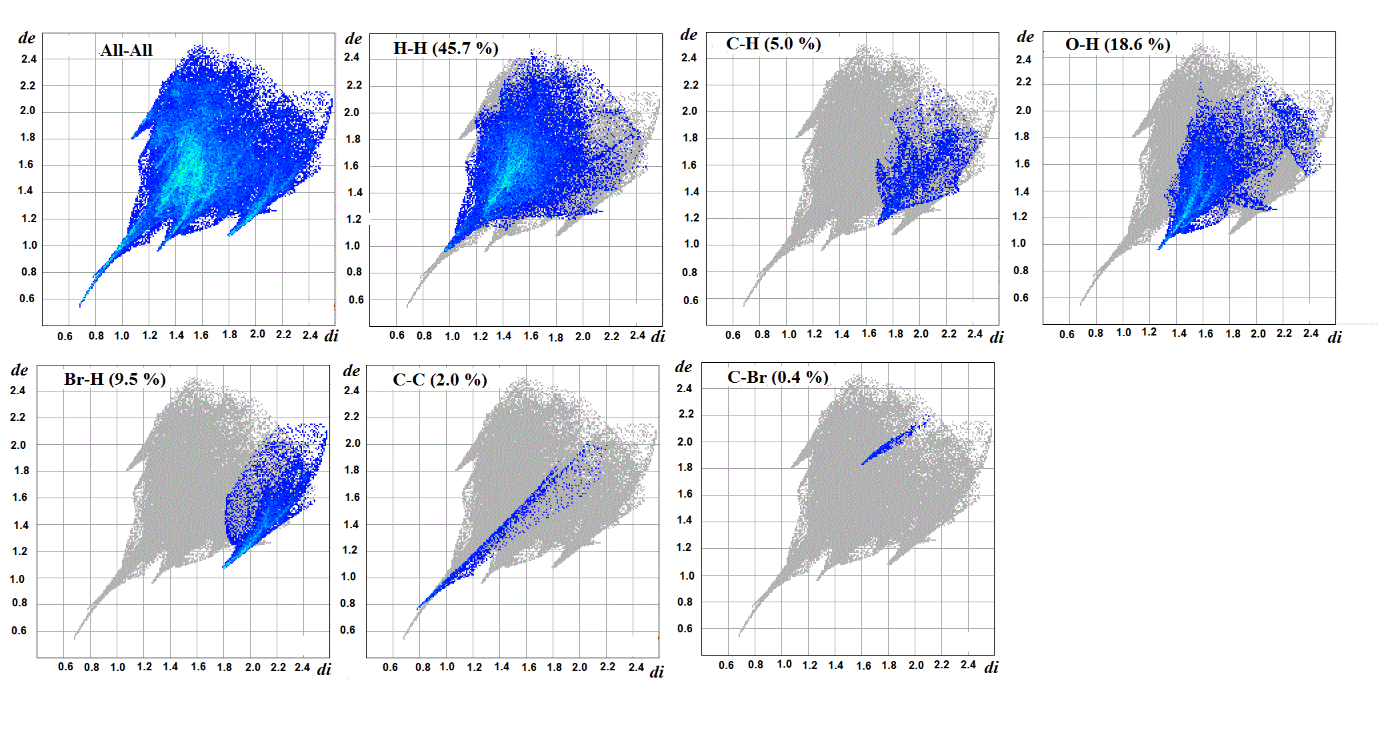


**(a)**


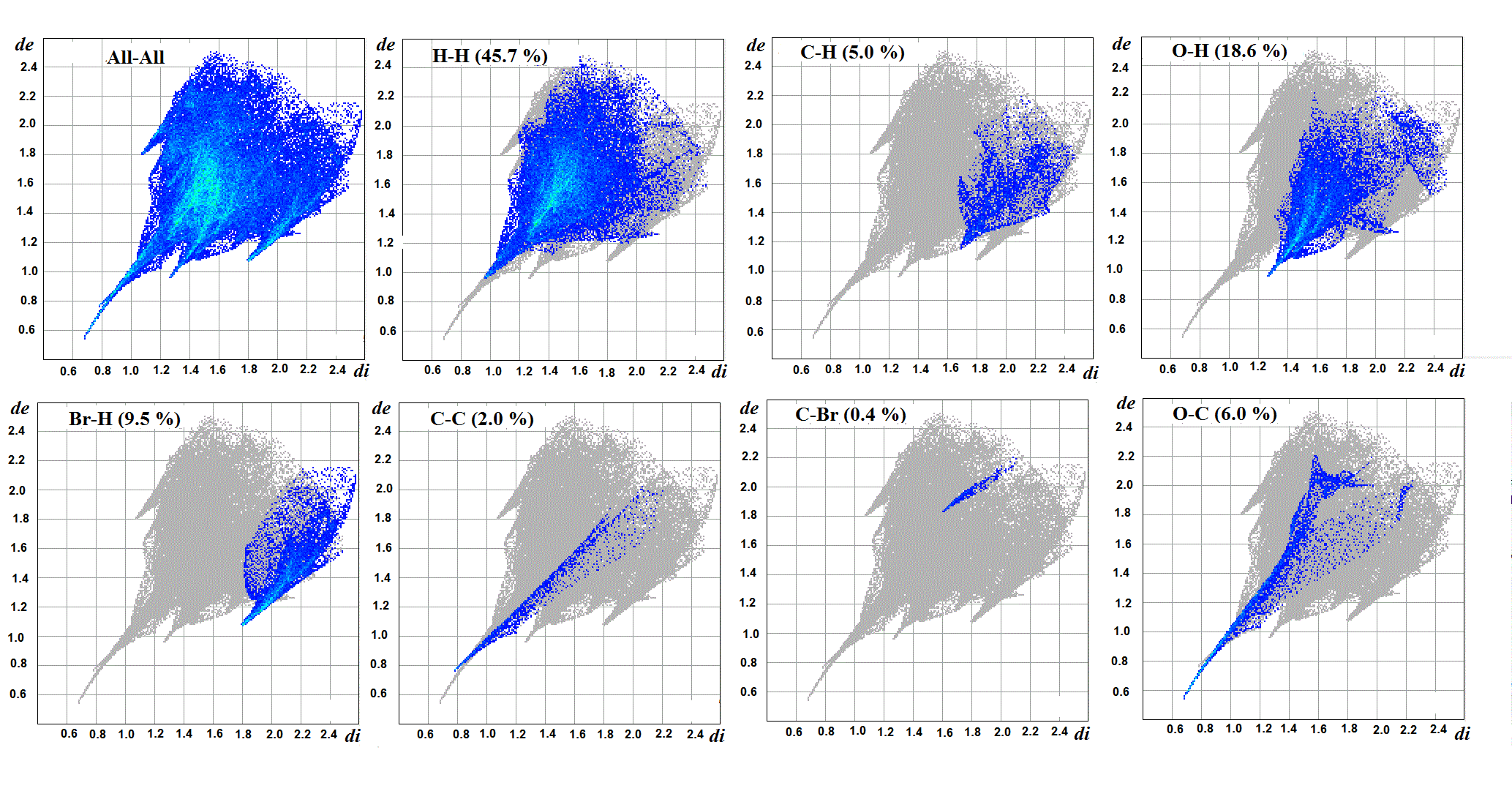


**(b)**

**Figure S5**: Two-dimensional fingerprint plots delineated into different contact types for complex **1.** Blue to cyan colour coding is applied to reflect an increasing density of overlapπng points in the plots. The grey background contours correspond to the plots integrated for all contact types.


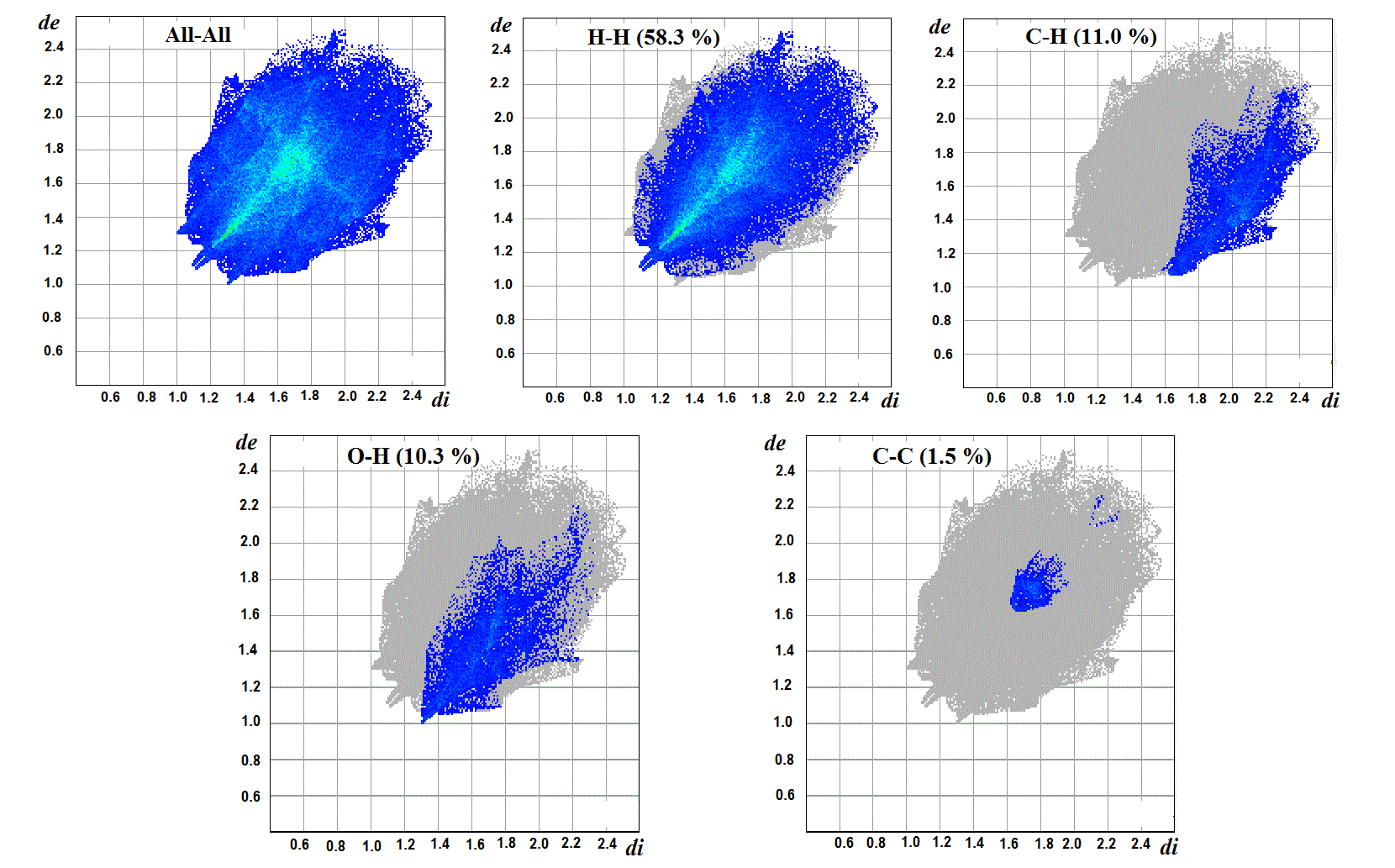


**(c)**


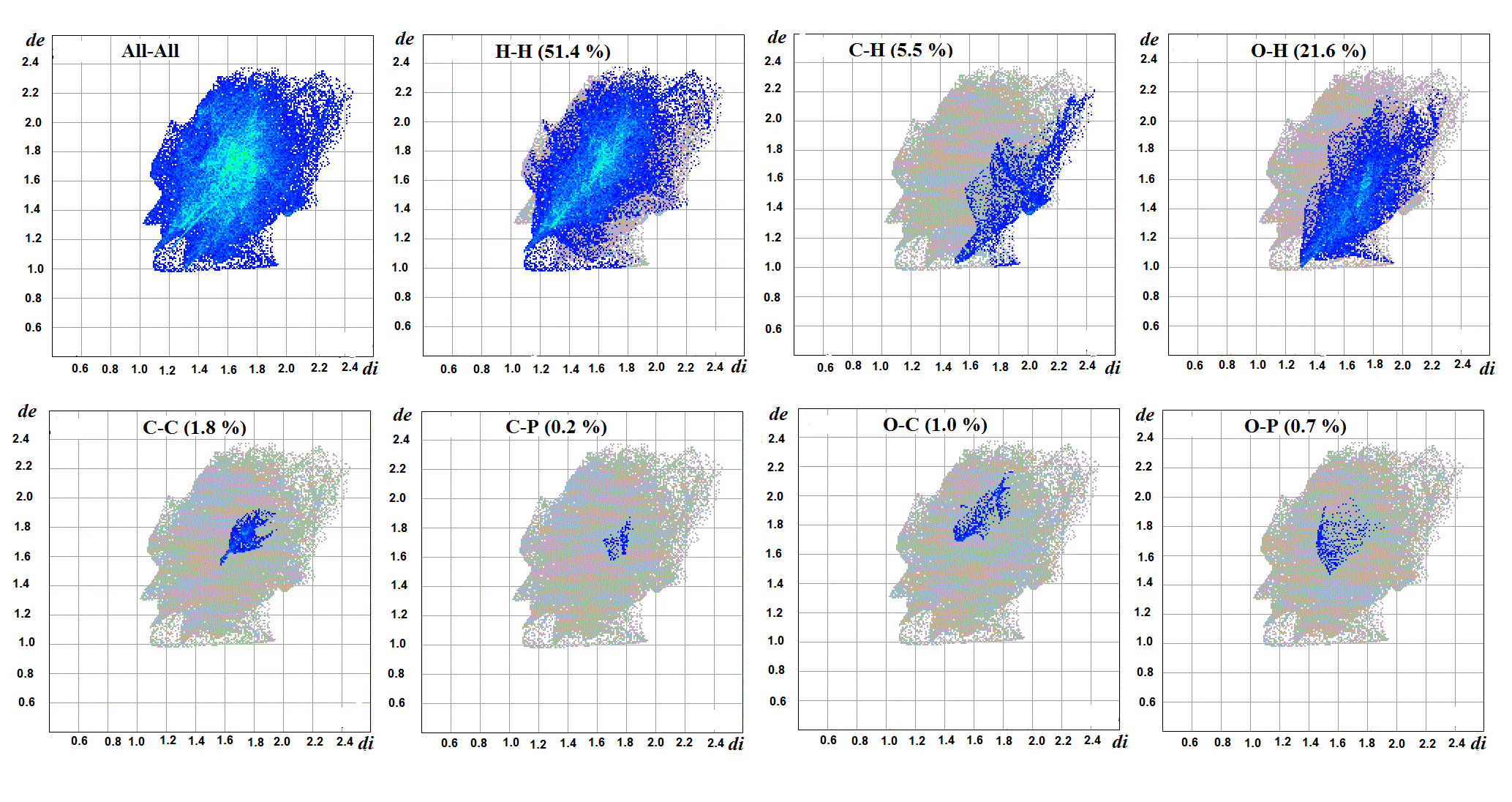


**(d)**

**Figure S6**: Two‐dimensional fingerprint plots delineated into different contact types for complex **2.** Blue to cyan colour coding is applied to reflect an increasing density of overlapπng points in the plots. The grey background contours correspond to the plots integrated for all contact types.


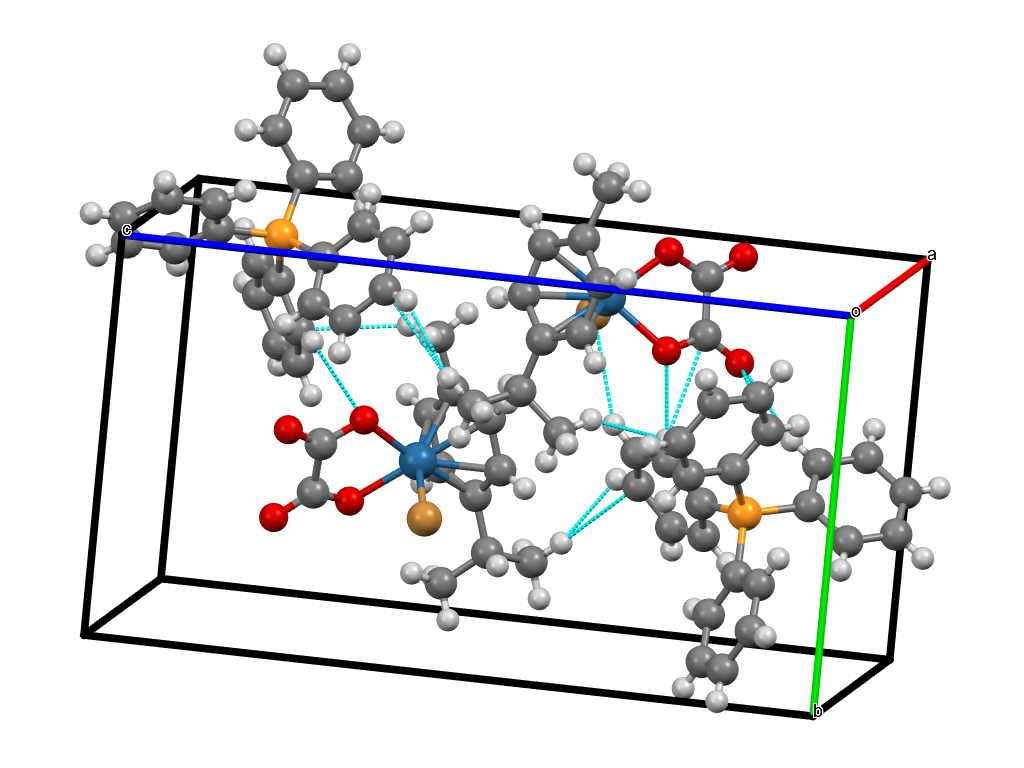


**Figure S7**: Packing diagram of **1** showing various types of interactions in a unit cell.


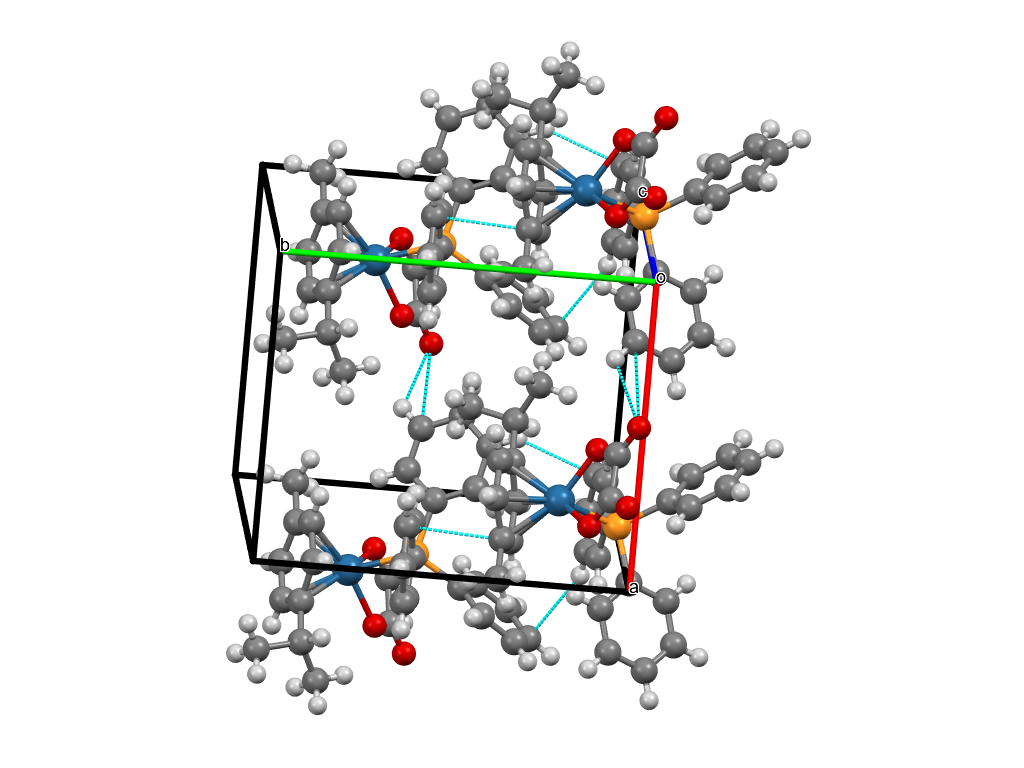


**Figure S8**: Packing diagram of **2** showing various types of interactions in a unit cell.

**Table S1**: Single crystal XRD data and DFT calculated geometrical parameters of **1** and **2**. [Selected Bond lengths (Å) and bond and torsion angles (°)].

|  | **XRD** | | **DFT (GGA/PBE)** | |
| --- | --- | --- | --- | --- |
| **Bond Lengths (Å)** | **1** | **2** | **1** | **2** |
| Os1−C1 | 2.163(6) | 2.201(4) | 2.187 | 2.231 |
| Os1−C2 | 2.175(6) | 2.234(4) | 2.219 | 2.253 |
| Os1−C3 | 2.165(5) | 2.245(4) | 2.188 | 2.255 |
| Os1−C4 | 2.184(6) | 2.208(3) | 2.225 | 2.254 |
| Os1−C5 | 2.150(6) | 2.194(3) | 2.161 | 2.233 |
| Os1−C6 | 2.182(6) | 2.210(3) | 2.218 | 2.263 |
| C1−C2 | 1.416(9) | 1.446(6) | 1.441 | 1.450 |
| C2−C3 | 1.414(12) | 1.387(5) | 1.433 | 1.414 |
| C3−C4 | 1.414(11) | 1.436(5) | 1.440 | 1.442 |
| C4−C5 | 1.415(10) | 1.418(5) | 1.435 | 1.431 |
| C5−C6 | 1.438(10) | 1.430(5) | 1.432 | 1.436 |
| C6−C1 | 1.417(9) | 1.407(5) | 1.439 | 1.425 |
| Os1−O3 | 2.099(4) | 2.093(3) | 2.114 | 2.098 |
| Os1−O1 | 2.100(4) | 2.078(3) | 2.093 | 2.081 |
| Os1−Br1 | 2.5324(7) | − | 2.572 | − |
| Os1−P1 | − | 2.3503(11) | − | 2.361 |
| Os−Centroid_cymene_ | 1.642 | 1.700 | − | − |
| **Bond Angles (°)** | | | | |
| O1–Os1–O3 | 77.05(16) | 77.77(10) | 77.299 | 78.254 |
| O3–Os1–Br1 | 85.19(13) | – | 85.429 | − |
| O1–Os1–Br1 | 84.34(12) | – | 85.807 | − |
| O3–Os1–P1 | – | 86.99(8) | − | 88.345 |
| O1–Os1–P1 | – | 88.67(8) | − | 86.127 |
| **Torsion Angles (°)** | | | | |
| O1–C11–C12–O3 | -1.04 | 3.7(5) | 0.090 | -0.538 |
| O2–C11–C12–O4 | -2.02 | 1.7(6) | -1.030 | -1.167 |

**UV-Vis spectra**

**Figure S9:** UV-Vis absorption spectrum of complex **1** in DMSO.

**Figure S10:** UV-Vis absorption spectrum of complex **2** in DMSO.

**Biochemical studies**

1. **Stability studies**

**Figure S11:** UV-Vis absorbance spectra, illustrating the stability study of complex **1** in DMSO and Tris buffer over 3 hours**.**

**Figure S12:** UV-Vis absorbance spectra, illustrating the stability study of complex **2** in DMSO and Tris buffer over 3 hours.

1. **DNA binding study**

**Figure S13:** Electronic spectra of complex **1** in Tris-HCl buffer upon adding various increments of CT-DNA solution over 3 hours. Arrow illustrates the hypochromic and bathochromic shift upon an increase in the DNA concentration.

**Figure S14:** Electronic spectra of complex **2** in Tris-HCl buffer upon adding various increments of CT-DNA solution over 3 hours. Arrow illustrates the hypochromic and bathochromic shift upon an increase in the DNA concentration.
