# Supplementary material for: Molecular Structure, Spectroscopic, Frontier Molecular Orbital Analysis, Molecular Docking Studies, and In Vitro DNA-Binding Studies of Osmium(II)-Cymene Complexes with Aryl Phosphine and Aryl Phosphonium Assemblies
Source: Bioinorg Chem Appl. 2024 May 29;2024:6697523. doi: 10.1155/2024/6697523 (PMC11152764; doi:10.1155/2024/6697523)

checkCIF/PLATON report

You have not supplied any structure factors. As a result the full set of tests cannot be run.

THIS REPORT IS FOR GUIDANCE ONLY. IF USED AS PART OF A REVIEW PROCEDURE FOR PUBLICATION, IT SHOULD NOT REPLACE THE EXPERTISE OF AN EXPERIENCED CRYSTALLOGRAPHIC REFEREE.

No syntax errors found.   CIF dictionary   Interpreting this report

Datablock: KC064

```
Bond precision:   C-C = 0.0086 A           Wavelength=0.71073

Cell:              a=7.5565(8)     b=10.7431(12)        c=19.144(2)
                   alpha=90        beta=100.154(4)      gamma=90
Temperature:       173 K

                 Calculated                         Reported
Volume           1529.8(3)                          1529.8(3)
Space group      P 21                               P 21
Hall group       P 2yb                              P 2yb
Moiety formula   C24 H20 P, C12 H14 Br O4 Os        C24 H20 P, C12 H14 Br O4 Os
Sum formula      C36 H34 Br O4 Os P                 C36 H34 Br O4 Os P
Mr               831.73                             831.71
Dx,g cm-3        1.806                              1.806
Z                2                                  2
Mu (mm-1)        5.566                              5.566
F000             816.0                              816.0
F000'            813.59
h,k,lmax         9,14,25                            9,14,25
Nref             7370[ 3878]                        7360
Tmin,Tmax        0.098,0.630                        0.180,0.716
Tmin'            0.046

Correction method= # Reported T Limits: Tmin=0.180 Tmax=0.716
AbsCorr = INTEGRATION

Data completeness= 1.90/1.00       Theta(max)= 28.000

R(reflections)= 0.0238( 7252)                       wR2(reflections)=
                                                    0.0623( 7360)

S = 1.092                  Npar= 391
```

```
The following ALERTS were generated. Each ALERT has the format
       test-name_ALERT_alert-type_alert-level.
Click on the hyperlinks for more details of the test.
```

Alert level C

```
PLAT221_ALERT_2_C Solv./Anion  Resd 2  C   Ueq(max)/Ueq(min) Range        5.5 Ratio
PLAT223_ALERT_4_C Solv./Anion  Resd 2  H   Ueq(max)/Ueq(min) Range        4.4 Ratio
PLAT242_ALERT_2_C Low    'MainMol' Ueq as Compared to Neighbors of         C8 Check
PLAT250_ALERT_2_C Large U3/U1 Ratio for Average U(i,j) Tensor ....        2.7 Note
PLAT342_ALERT_3_C Low Bond Precision on  C-C Bonds ...............     0.0086 Ang.
PLAT411_ALERT_2_C Short Inter H...H Contact  H2       ..H8       .       2.12 Ang.
                                        1-x,-1/2+y,1-z  =      2_646 Check
```

Alert level G

```
PLAT232_ALERT_2_G Hirshfeld Test Diff (M-X)  Os1      --Br1      .       15.3 s.u.
PLAT883_ALERT_1_G No Info/Value for _atom_sites_solution_primary .     Please Do !
PLAT933_ALERT_2_G Number of HKL-OMIT Records in Embedded .res File          3 Note
PLAT967_ALERT_5_G Note: Two-Theta Cutoff Value in Embedded .res ..       56.0 Degree
```

```
   0 ALERT level A = Most likely a serious problem - resolve or explain
   0 ALERT level B = A potentially serious problem, consider carefully
   6 ALERT level C = Check. Ensure it is not caused by an omission or oversight
   4 ALERT level G = General information/check it is not something unexpected

   1 ALERT type 1 CIF construction/syntax error, inconsistent or missing data
   6 ALERT type 2 Indicator that the structure model may be wrong or deficient
   1 ALERT type 3 Indicator that the structure quality may be low
   1 ALERT type 4 Improvement, methodology, query or suggestion
   1 ALERT type 5 Informative message, check
```

Validation response form

Please find below a validation response form (VRF) that can be filled in and pasted into your CIF.

```
# start Validation Reply Form
_vrf_PLAT221_KC064
;
PROBLEM: Solv./Anion  Resd 2  C   Ueq(max)/Ueq(min) Range        5.5 Ratio
RESPONSE: ...
;
_vrf_PLAT223_KC064
;
PROBLEM: Solv./Anion  Resd 2  H   Ueq(max)/Ueq(min) Range        4.4 Ratio
RESPONSE: ...
;
_vrf_PLAT242_KC064
;
PROBLEM: Low    'MainMol' Ueq as Compared to Neighbors of         C8 Check
RESPONSE: ...
;
_vrf_PLAT250_KC064
```

```
;
PROBLEM: Large U3/U1 Ratio for Average U(i,j) Tensor ....        2.7 Note
RESPONSE: ...
;
_vrf_PLAT342_KC064
;
PROBLEM: Low Bond Precision on  C-C Bonds ..............     0.0086 Ang.
RESPONSE: ...
;
_vrf_PLAT411_KC064
;
PROBLEM: Short Inter H...H Contact  H2       ..H8       .       2.12 Ang.
RESPONSE: ...
;
# end Validation Reply Form
```

It is advisable to attempt to resolve as many as possible of the alerts in all categories. Often the minor alerts point to easily fixed oversights, errors and omissions in your CIF or refinement strategy, so attention to these fine details can be worthwhile. In order to resolve some of the more serious problems it may be necessary to carry out additional measurements or structure refinements. However, the purpose of your study may justify the reported deviations and the more serious of these should normally be commented upon in the discussion or experimental section of a paper or in the "special_details" fields of the CIF. checkCIF was carefully designed to identify outliers and unusual parameters, but every test has its limitations and alerts that are not important in a particular case may appear. Conversely, the absence of alerts does not guarantee there are no aspects of the results needing attention. It is up to the individual to critically assess their own results and, if necessary, seek expert advice.

Publication of your CIF in IUCr journals

A basic structural check has been run on your CIF. These basic checks will be run on all CIFs submitted for publication in IUCr journals (*Acta Crystallographica*, *Journal of Applied Crystallography*, *Journal of Synchrotron Radiation*); however, if you intend to submit to *Acta Crystallographica Section C* or *E* or *IUCrData*, you should make sure that full publication checks are run on the final version of your CIF prior to submission.

Publication of your CIF in other journals

Please refer to the *Notes for Authors* of the relevant journal for any special instructions relating to CIF submission.

PLATON version of 09/05/2022; check.def file version of 21/03/2022

Datablock KC064 - ellipsoid plot

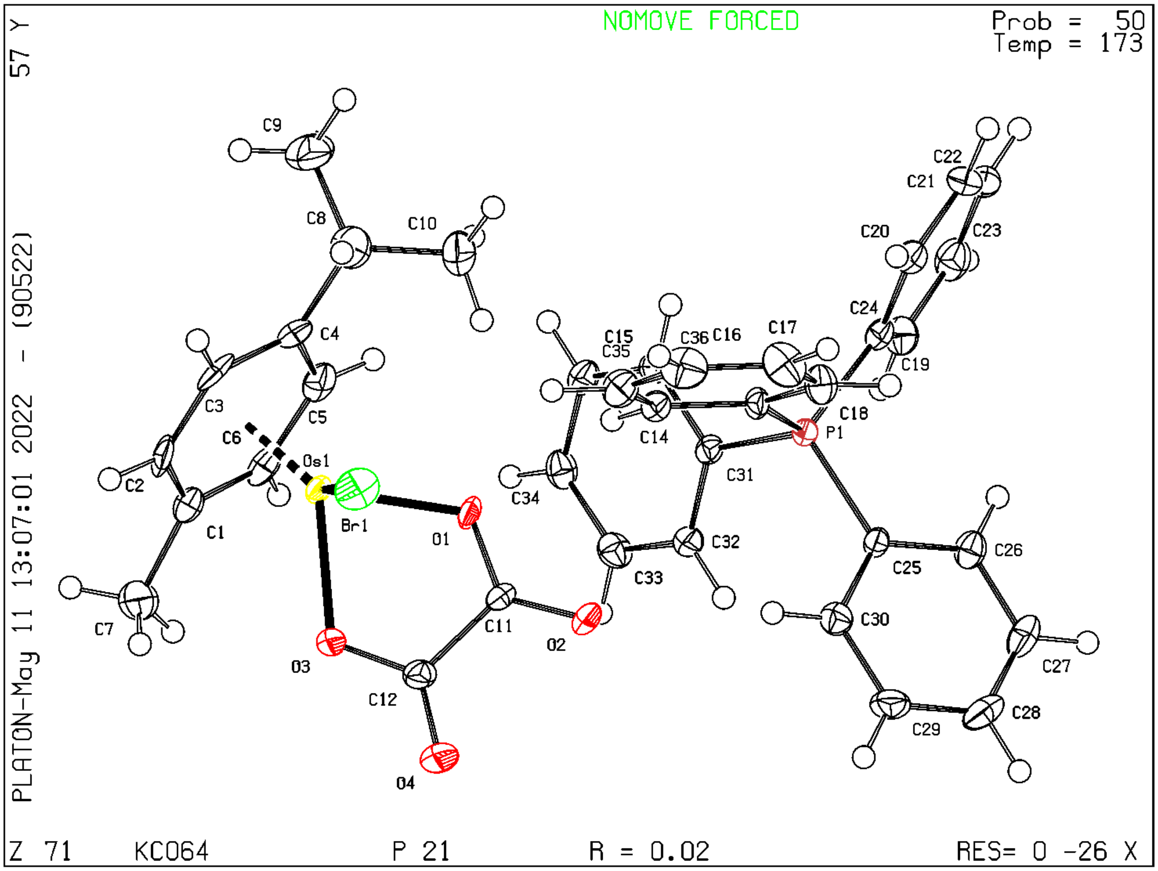

Supplement: Supplementary Materials — Vibrational spectroscopy data. Figure S1: FT-IR data of 1 from 2000 to 400 cm−1. Figure S2: FT-IR data of 2 from 2000 to 400 cm−1. Figure S3: FT-Raman spectrum of 1 from 2000 to 0 cm−1. Figure S4: Raman spectrum of 2 from 2000 to 0 cm−1. Hirshfeld Surface Analysis. Figure S5: Two-dimensional fingerprint plots portrayed into various contact types for complex 1. Figure S6: Two‐dimensional fingerprint plots portrayed into various contact types for complex 2. Figure S7: Packing diagram of 1 showing various types of interactions in a unit cell. Figure S8: Packing diagram of 2 showing various types of interactions in a unit cell. Table S1: Single-crystal XRD information and DFT-calculated geometrical parameters of 1 and 2 [Selected bond lengths (Å) and bond and torsion angles (°)]. DNA-Binding Study. Figure S9: UV-Vis absorption data of 1 in DMSO. Figure S10: UV-Vis absorption data of 2 in DMSO. Figure S11: UV-Vis absorbance spectra, illustrating the stability study of 1 in DMSO and Tris buffer over 3 hours. Figure S12: UV-Vis absorbance spectra, illustrating the stability study of 2 in DMSO and Tris buffer over 3 hours. Figure S13: Electronic data of 1 in Tris-HCl buffer following addition of various increments of CT-DNA solution over the period of 3 hours. Figure S14: Electronic data of 2 in Tris-HCl buffer following addition of various increments of CT-DNA solution over the period of 3 hours. X-ray crystallography. CheckCIF/PLATON details for the crystal structure solving of 1 and 2. [file 6697523.f1.zip › CheckCIF Complex 1.pdf]
